# snpQT: flexible, reproducible, and comprehensive quality control and imputation of genomic data

**DOI:** 10.12688/f1000research.53821.2

**Published:** 2021-11-29

**Authors:** Christina Vasilopoulou, Benjamin Wingfield, Andrew P. Morris, William Duddy

**Affiliations:** 1 Northern Ireland Centre for Stratified Medicine, University of Ulster, Derry/Londonderry, BT47 6SB, UK; 2 Centre for Personalised Medicine, University of Ulster, Derry/Londonderry, BT47 6SB, UK; 3 Centre for Genetics and Genomics Versus Arthritis, Centre for Musculoskeletal Research, University of Manchester, Manchester, UK

**Keywords:** GWAS, Quality Control, GWAS pipeline, Nextflow, Imputation, SNPs, Genomic Variants, BioContainers, QC, Population Stratification, GWAS, Anaconda

## Abstract

Quality control of genomic data is an essential but complicated multi-step procedure, often requiring separate installation and expert familiarity with a combination of different bioinformatics tools. Software incompatibilities, and inconsistencies across computing environments, are recurrent challenges, leading to poor reproducibility. Existing semi-automated or automated solutions lack comprehensive quality checks, flexible workflow architecture, and user control. To address these challenges, we have developed snpQT: a scalable, stand-alone software pipeline using nextflow and BioContainers, for comprehensive, reproducible and interactive quality control of human genomic data. snpQT offers some 36 discrete quality filters or correction steps in a complete standardised pipeline, producing graphical reports to demonstrate the state of data before and after each quality control procedure. This includes human genome build conversion, population stratification against data from the 1,000 Genomes Project, automated population outlier removal, and built-in imputation with its own pre- and post- quality controls. Common input formats are used, and a synthetic dataset and comprehensive online tutorial are provided for testing, educational purposes, and demonstration. The snpQT pipeline is designed to run with minimal user input and coding experience; quality control steps are implemented with numerous user-modifiable thresholds, and workflows can be flexibly combined in custom combinations. snpQT is open source and freely available at https://github.com/nebfield/snpQT. A comprehensive online tutorial and installation guide is provided through to GWAS (https://snpqt.readthedocs.io/en/latest/), introducing snpQT using a synthetic demonstration dataset and a real-world Amyotrophic Lateral Sclerosis SNP-array dataset.

## Introduction

Genome-Wide Association Studies (GWAS) seek to identify genetic variants that have a statistically significant association to a trait, such as a disease or other phenotype of interest. GWAS has been widely employed towards a large variety of objectives, including genetic epidemiology, precision medicine, polygenic risk scores, therapeutic development, network-based and machine learning approaches ^1– 6^. A rapid explosion in the quantity of genomic data has created the need for systematic, standardised, and reproducible quality control (QC). Assuring high quality of genomic data is necessarily a complex multi-step procedure with multiple challenges, but it is essential in order to ensure reproducible and reliable results ^1, 7– 9^. Although there are well-established steps and good practices ^10, 11^, there is no standardised and universally followed workflow, which impacts on the reproducibility and comparability of results ^7, 9^.

Existing approaches, including semi-automated tools ^12^, can involve a time-consuming "trial and error" approach, requiring the analyst to check the distributions of parameters in plots produced over many rounds of adjustments, and to manually enter commands in a long list of QC steps one-by-one, or in a series of shell scripts. The analyst may encounter incompatibility and scalability problems, installation difficulties as well as spending valuable time familiarizing themselves with a number of different tools that sometimes lack detailed documentation. Software architecture tools such as nextflow and BioContainers can address these issues ^13^ and have been proposed as automated solutions ^14^; however restrictions exist in terms of limited and relatively rigid QC analysis, lacking such steps as imputation, limited variety of threshold choice and plot outputs, and the requirement for users to have extensive knowledge of the software in order to tailor their analysis.

**Figure 1.**
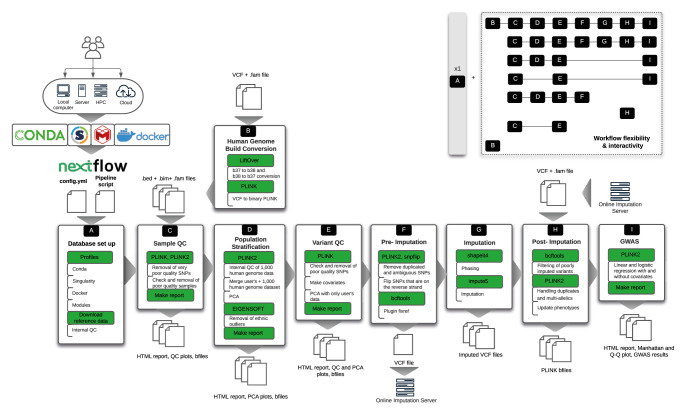
Outline of the snpQT architecture, which includes nine core workflows (A–I) that are implemented using nextflow. To provide a reproducible and scalable pipeline, snpQT automatically loads software dependencies using Anaconda, Singularity, Docker, or environment modules. This versatility of available implemented profiles provides the opportunity to the user to run snpQT in a variety of environments ranging from a local laptop to HPC. Each workflow expects specific inputs either from the user or from the outputs generated by other workflows. Main tools and key processes (modules) are highlighted in green. Examples of different workflow combinations are represented in the upper-right corner, showing the flexibility and interactivity among the implemented workflows. HPC: High Performance Computing cluster, VCF: Variant Call File; QC: Quality Control; PCA: Principal Component Analysis; GWAS: Genome-Wide Association Studies.

Here we present snpQT (shown in Figure 1): a standardised, flexible, scalable, automatic pipeline tool that provides comprehensive quality control, with imputation and association analysis, including publication-ready figures for data interpretation and validation for every QC step. Most computing environments running Linux are supported, including standalone devices and High Performance Computing (HPC) clusters. In addition, a flexible architecture enables various workflow combinations (as shown in Figure 1). The majority of implemented thresholds and QC steps are user-modifiable at run-time, both from the command line and in a user-created parameter file. Detailed reports, including distribution plots both before and after applying each QC threshold, aid the user in decision-making and it is easy to re-run an analysis with modified thresholds to arrive at optimal output.

## Methods

### Implementation

snpQT was developed as a set of nine core workflow components implemented with the nextflow workflow management system ^13^. Each workflow component can be executed using container engines (Docker or Singularity) or environment managers (Anaconda or Modules). Execution is controlled by profiles. Container engines use standardised BioContainers ^15^ and environment managers use BioConda environments by default. Combining independent containerised modules into workflows, and providing multiple workflow combinations, using the nextflow architecture, enables snpQT to be a reproducible and uniquely versatile tool for the analysis of human variant genomic data. Containers improve end-user experience and promote reproducible research by automatically provisioning bioinformatics software as required and improving numerical stability ^13, 16^. Running individual modules in independent containers also solves a common problem when installing potentially incompatible software packages ^17^. In addition, nextflow enables caching at continuous checkpoints, so users can alter thresholds without needing to rerun earlier parts of the analysis. Briefly, if a module has the same input and parameters that a previous pipeline run has already processed, then the cached work is passed to the next module in the workflow instead of recomputing new work. This means that if a user runs multiple jobs and changes parameters at a later stage in the overall pipeline, then earlier unchanged stages are skipped, saving time.

As each genomic study is unique, this requires a tailored and flexible pipeline with informative representations of intermediate quality control data. snpQT is designed to offer multiple combinations of workflows as well as modifiable threshold parameters for multiple steps (as shown in Figure 1). Workflow A runs only once, performing a local database set up, downloading and preparing reference files ^18, 19^ and setting up specific versions of tools using Anaconda, Singularity, Docker or Environment Modules. Workflow B has been created for the user to remap their genomic dataset from build 38 to 37 and vice versa. Workflow C performs Sample QC, including checks for missing call rate, sex discrepancies, heterozygosity, cryptic relatedness, and missing phenotypes. Workflow D performs Population Stratification: after an internal QC of the reference genome and user’s dataset, the two datasets are merged and prepared for the automatic removal of outliers using EIGENSOFT ^20^. Principal Component Analyses are carried out before and after the outlier removal. Workflow E performs the main Variant QC, checking missing call rate, Hardy-Weinberg equilibrium deviation, minor allele frequency, missingness in case/control status, and generates covariates for GWAS, based on a user-modifiable number of Principal Components (or users may provide a covariates file). Workflow F is for pre-imputation quality control, preparing the dataset for imputation, while Workflow G performs local phasing and imputation using shapeit4 ^21^ and impute5 ^22^. Workflow H performs post-imputation QC where poorly imputed variants are removed, different categories of duplicated variant IDs are handled and the phenotypes of the dataset are updated. The workflows’ structure also allows for users to upload their data to an external imputation server, or use a different reference panel. Workflow I performs GWAS with and without adjustment of covariates (if the covariates are not provided by the user, snpQT uses the first five Principal Components generated from Population Stratification in Workflow D), outputting summary statistics, along with a Manhattan plot and a Q-Q plot.

As it can be challenging to choose the correct threshold for a metric, snpQT provides a "Make Report" module in each of the main Workflows C, D, E, and I, that provides interactive HTML reports summarising all the plots for both before and after the chosen thresholds have been applied, enabling the user to easily inspect and check if the chosen thresholds are appropriate following each run of the analysis – and to re-run with modified thresholds as needed. Detailed summary logs and graphs are also provided throughout, depicting the total number of samples and variants in each step, for users who need easy and fast inspection of the processes, as well as for users who want a more in-depth report prompting users towards the locations of intermediate files and logs.

### Operation

snpQT is implemented in nextflow, R and Unix command line utilities. The minimum software requirements to run snpQT are Java 8, nextflow v21.04.3, and a POSIX-compatible operating system (tested on CentOS 8). The hardware requirements scale with input data and workflows: typically quality control checks require less than 16GB of RAM and 4 cores on large datasets of 40,000 individuals. However, imputation requires significant computing power - up to 50GB of RAM per chromosome per core. As well as those already listed, the following tools are used: picard ( https://broadinstitute.github.io/picard/), PLINK ^23^, PLINK2.0 ^24^, samtools ^25^, and snpflip ( https://github.com/biocore-ntnu/snpflip).

The latest release of the 1,000 Genomes Project data ^18^ is used as a reference panel in both VCF and processed PLINK2 formats ^19^. A part of the population stratification and variant QC implementation was inspired by the work of Marees *et al.* ^11^. Optional software requirements include Anaconda, Singularity, Docker and Environment Modules which provide a simple method to install and run the underlying collection of bioinformatics software described above without worrying about software inconsistencies or incompatibilities and without need for manual installation:

- Anaconda is suitable for users who are not interested in performing local imputation and who do not have root access in their machines. Users can still run pre-imputation and post-imputation QC, as well as all the remaining QC-related workflows of snpQT.
- Singularity ^26^ automatically provisions containers to run software packages, while supporting all the snpQT implemented workflows. Singularity provides the user with full scalability, supporting even HPC environments.
- Docker requires root access, which enables the installation of impute5, which is used for imputation (root access is not required for running the analysis if Singularity is used).
- Environment Modules are useful to run all stages of the pipeline in HPC environments, where root access is not available, but require some user configuration because installed packages and package names are custom to each HPC environment.

Full documentation of snpQT, including an installation guide, a Quickstart explanation of workflow combinations and commands, a complete description of workflows, and an in-depth tutorial are provided at https://snpqt.readthedocs.io/en/latest/. The following Use Case section gives examples of input and output with explanatory context, and explains all of the key parameters needed to make use of snpQT.

## Use case

This section provides a guide through the snpQT Quality Control pipeline, explaining the steps and demonstrating the application of the tool, using a synthetic dataset which is free and available with the tool. The plots shown are based on this synthetic dataset, which has some artificial structure but is adequate for demonstration purposes. The online tutorial includes plots with natural distributions, derived from a real-world Amyotrophic Lateral Sclerosis dataset of 2,000 samples (1,000 cases and 1,000 controls) taken from a restricted-access dbGaP project ^27^.

### Installation

Before downloading and running snpQT, depending on their needs, the user should have downloaded nextflow (v21.04.3 or later) and any of the following software including Anaconda, Singularity, Docker or Environment Modules. To begin installation, the latest release of the repository can be cloned and set up can be initiated by running the following command (future users should check the GitHub repository at https://github.com/nebfield/snpQT/releases for the latest release number):


git clone --branch v0.1.7 https://github.com/nebfield/snpQT.git


Before starting to use any of the implemented workflows, it is necessary to set up a local database of reference and auxiliary files that snpQT requires to run. Because of the large volume of reference data in imputation workflow, we have designed two types of database. The first is the core reference database, which is sufficient for the human genome build conversion, sample and variant quality control, population stratification, pre-imputation, post-imputation, and GWAS workflows. The second database is required only for local imputation, and downloading the latest release of the 1,000 Genomes Project data. In either case, it is necessary to first set up the core database. An already processed `.tar.gz` file (17.3GB, when unzipped it requires 19.7GB of space) of the required reference data can be downloaded using the following commands:


cd snpQT
mkdir db
wget 'https://zenodo.org/record/4916469/files/core.tar.gz?download=1' -O db/core.tar.gz
cd db && tar -xvf core.tar.gz



The above command will download the core reference files and store them in a db/ folder in the snpQT directory.

If the user is interested in local imputation then the following command should also be run (in addition to the previous commands for the core database set up):


wget 'https://zenodo.org/record/4916469/files/impute.tar.gz?download=1' -O impute.tar.gz
tar -xvf impute.tar.gz --strip-components=1


This command will download an already processed .tar.gz (13GB, when unzipped it requires 15GB of space) imputation reference file.

After the download of the local database, the user should select a combination of two profile settings. The choice of these depends on the system environment and the analysis requirements. Firstly, there are two options that control where snpQT modules can be run:

-profile standard: suitable for users who wish to run snpQT on a local computer.
-profile cluster: suitable for users who wish to run snpQT on HPC, using a SLURM scheduler.

The second profile setting concerns the way in which specific versions of bioinformatics tools and libraries are installed. To accommodate a variety of usage needs we provide four different options:

-profile conda: uses Anaconda to automatically install software packages. Suitable for users who are not interested in imputation modules.
-profile singularity: uses Singularity to automatically provision containers to run software packages. Singularity enables the user to run every snpQT module, while providing total scalability.
-profile docker: uses Docker to automatically install software packages. Docker requires root access to build and run containers but enables to run every snpQT module.
-profile modules: uses Environment Modules to automatically provision containers to run software packages. Environment Modules and Singularity are useful for cluster environments.

The user should select one from among the first two profile settings, and one from among the other four settings. For example -profile standard,conda or -profile cluster,singularity.

The snpQT workflows generate many intermediate files that are not shown directly to the user, and are usually not needed by the user. Nextflow stores these in the snpQT/work/ directory by default. snpQT will remember previous work that it has done, such that it may automatically avoid needless repetition of previously completed work if asked to run a different stage of the pipeline, or to run again with tweaked parameters, on the same input data. When the work/ folder is deleted, all work for the analysis will need to be redone.

### Datasets

After the download and set up of the database and the profiles, the user can start exploring any of the implemented snpQT workflows. A synthetic demonstration dataset is available with snpQT, which can be used to gain familiarity with the workflows and modules, while ensuring reproducible results identical to those shown in this section. The synthetic dataset is located within the data/ folder, and consists of a .vcf.gz file and three binary PLINK files (.bed, .bim and .fam). The dataset contains 6,517 randomised genotypes of chromosome 1, derived from 100 female samples having balanced binary phenotypes (i.e. 51 cases vs 49 controls). The chromosome positions, alleles and SNP IDs have been updated according to human 1,000 Genomes Project data (hg37).

Below, we provide an exploration of snpQT's functionalities. We hypothesize a simple scenario where the user wishes to run snpQT in a local computer and is interested in both quality control workflows and imputation. In this scenario, the user should more likely choose -profile standard,singularity. However, the tutorial is also useful for users who wish to run their experiments in a HPC cluster, using -profile cluster,singularity instead. The user can modify all the implemented parameters in the form of a file using -params-file parameters.yaml, or can provide parameter settings as flags on the command line. The preferred way to run snpQT is to use only a parameter file, as this acts as a concise record of settings chosen per job, enables the user to consider all settings in one place, and keeps the terminal clean. We provide an example parameter file in our online documentation at https://snpqt.readthedocs.io/en/latest/quickstart/parameters/ and at snpQT’s GitHub repository. However, for the purpose of this tutorial we use flags in combination with a parameter file to highlight some of the parameters in use for each example.

### Human genome build conversion

The Human Genome Build Conversion workflow converts genomic files aligned in build 38 to build 37 and vice versa, using Picard’s LiftoverVcf utility. snpQT assumes that your input genomic data are aligned to build 37, as some of the workflows are designed to accept this input. Despite that GRCh37 human reference genome is not the most recent one, it is the most frequently used build among current public reference genomic datasets (e.g. 1,000 Genomes data, Haplotype Reference Consortium panel), online imputation servers (e.g. Sanger Imputation Server) and available SNP-array datasets, and for this reason snpQT has been designed to support only GRCh37 (hg19) for some workflows. Hence, this workflow can be helpful for users with data aligned to build 38 to convert them to build 37, in order to run QC and population stratification. The workflow may also be helpful when a user has finished their main QC and wishes to upload their data to an external imputation server that uses a reference panel aligned in b38 (i.e. TOPMed) or b37 (i.e. Haplotype Reference Consortium panel), or to perform a local imputation using a reference panel which is aligned in another build.

The genomic build of the synthetic dataset (which is aligned to b37) can be converted to b38 by running the following code, with input files and options explained below:


nextflow run main.nf \
  -profile standard,singularity \
  -resume \
  -params-file parameters.yaml \
  --vcf data/data/toy.vcf.gz \
  --fam data/data/toy.fam \
  --convert_build \
  --input_build 37 \
  --output_build 38 \
  --results convert_build_toy/

- Input files:
  ○ ––vcf: This workflow requires a valid VCF file of human genomic data.
  ○ ––fam: This workflow also requires an accompanying PLINK .fam file which should contain the same samples as the VCF file.
- snpQT options:
  ○ ––convert_build: runs the build conversion workflow.
  ○ ––input_build: defines the build of the input data [37/38].
  ○ ––output_build: defines the build of the output data [37/38].
  ○ ––mem: assigns the memory size that the LiftoverVCF utility can use.
  ○ ––results: specifies the directory where the output files are stored. To retain results from separate analyses, the name of the results folder should be changed between runs.
- Nextflow options:
  ○ –resume: runs multiple jobs using cached files (so skipping processes which are not affected by new changes). When running a different stage of the pipeline on the same input data, this will cause snpQT to avoid needless repetition of work already done.
  ○ –profile standard,singularity can be replaced using any of the other profile choices we provide depending on your installation and needs e.g. whether local imputation is needed or not ( -profile standard,[docker/conda]), and if you wish to run your experiments in a HPC cluster ( -profile cluster,[singularity/modules]).
  ○ -params-file accepts a parameter file including a list of modified parameters. We provide an example parameter file in our GitHub repository and in our online documentation at https://snpqt.readthedocs.io/en/latest/quickstart/parameters/.

When this work is run successfully, a new folder will be created named convert_build_toy/ which contains a files/ sub-folder with three binary PLINK files (with updated genomic information, and phenotypic information preserved), and also a .vcf.gz file for users who prefer this format for other purposes.

### Main quality control

snpQT’s main Quality Control analysis is divided into two distinct nextflow workflows: Sample and Variant QC. Sample and Variant QC can be run using the parameter –-qc. The required input files are binary PLINK files which can be imported using the parameters –-bed, –-bim and –-fam, for .bed, .bim and .fam PLINK files, respectively. The main checks of Sample QC are listed below, with accompanying parameters and explanation:

- **Missing variant call rate check:** Remove very poor quality SNPs based on call rate. These SNPs will be removed anyway at the variant QC stage, and applying the filter here avoids unnecessary removal of samples that may otherwise be of good quality.
- **Missing sample call rate check:** Remove samples with lower than a user-specified percentage call rate using the –-mind parameter. The distribution for all samples using histograms and scatterplots is visualised before and after the applied threshold.
- **Check for sex discrepancies:** Remove problematic samples for which (1) pedigree sex does not agree with the predicted sex based on sex chromosome homozygosity or (2) there is no sex phenotype present in the .fam file. This step can be skipped by setting the –-sexcheck false parameter (this modification could be useful for users whose data do not contain any sex chromosomes).
- **Removal of non-autosomal SNPs:** If the user wishes to remove the sex chromosomes, the –-keep_sex_chroms false parameter can be set.
- **Heterozygosity check:** Identify and remove heterozygosity outliers (samples that deviate more than 3 units of standard deviation from the mean heterozygosity). The distribution of the samples’ heterozygosity is visualised by a histogram and a scatterplot. Extreme heterozygosity implies inbreeding and/or DNA contamination. This step can be skipped using the –-heterozygosity false parameter.
- **Check for cryptic relatedness and duplicates:** Check for cryptic pairs of relatives or duplicated samples using PLINK2’s relationship-based pruning. You can control the level of accepted relatedness using the --king_cutoff parameter.
- **Removal of samples with a missing phenotype:** Remove samples with missing phenotypes using the parameter –-rm_missing_pheno true. As *missing phenotype* here we refer to phenotype status (i.e. the last column in the PLINK .fam file).

The second part of the main QC is Variant QC, which is again implemented using the --qc parameter. It is considered good practice to first filter poor quality samples in order to reduce the risk of removing a potentially high-risk variant during Variant QC. For this reason, the Population Stratification workflow (if chosen to be run by the user, as explained in the next subsection), which is essentially a Sample QC step, is designed to run between Sample QC and Variant QC, as seen in Figure 1. The Variant QC module contains the following steps:

- **Missing variant call rate check**: Remove poor quality SNPs using the parameter --variant_geno.
- **Hardy-Weinberg equilibrium (HWE) deviation check**: Remove SNPs that significantly deviate from the Hardy-Weinberg equilibrium (HWE), indicating a genotyping error, and visualise the distribution of SNPs with extreme deviation. The p-value threshold can be controlled using the parameter --hwe.
- **Minor Allele Frequency (MAF) check**: Remove SNPs with low MAF and visualise the distribution. The threshold can be modified using the --maf parameter in snpQT. Rare SNPs (having a very low MAF) are usually considered as false positives and need to be excluded from further analysis.
- **Missingness in case/control status check**: Remove SNPs with a statistically significant association of missingness (low call rate) and case/control status. The threshold can be modified using the parameter --missingness or by setting missingness to true in the parameter file. This check cannot be performed for quantitative data. For this reason, if the user’s data are not binary, the --linear true parameter can be used to skip this check.
- **Generate covariates using the first X Principal Components of each sample**: Perform Principal Component Analysis and visualise the 2D and interactive 3D PCA plots annotating the samples by the phenotype status. If the GWAS workflow is called (using the parameter --gwas, or setting gwas to true in the parameter file), the first X Principal Components (PCs) are used to account for inner population structure. The number of PCs (the value of X) can be adjusted using the --pca_covars parameter which can take as input a number from 1 to 20, with 1 starting from the first Principal Component of the PCA. Prior to the PCA, snpQT keeps only independent markers performing variant pruning using PLINK. This behaviour can be controlled using the parameter --indep_pairwise.

The synthetic toy dataset does not contain sex chromosomes, so to avoid PLINK producing an error it is important to add --sexcheck false to the command line or sexcheck: false to the parameter file, in order to skip the step which checks for sex discrepancies. Main QC on the toy dataset can be performed by running the following command:


nextflow run main.nf \
  -profile standard,singularity \
  -resume \
  -params-file parameters.yaml \
  --bed data/toy.bed \
  --bim data/toy.bim \
  --fam data/toy.fam \
  --qc \
  --results results_toy/ \
  --sexcheck false


On completion, the results_toy/ directory will now contain as many folders as the number of workflows that were run. Based on the example given, a results_toy/qc/ folder should be present, containing the following sub-folders and files (this structure will be similar for most other workflows):

- A bfiles/ folder including the binary PLINK files of the last step of the corresponding module.
- A figures/ folder including all generated plots for the steps that have been run within this workflow, as well as log plots summarising the number of samples and variants in each step of the workflow.
- A logs/ folder including two .txt files (sample_qc_log.txt and variant_qc_log.txt) summarising details about the numbers of samples, variants, and phenotypes for each step of Sample and Variant QC, as well as the working directory where the intermediate files for each process are stored, so that it is easier for the user to inspect the results.
- Two HTML reports for Sample and Variant QC summarising all the "before-and-after the threshold" plots generated in each step, as well as a plot demonstrating the number of samples and variants in every step.

**Figure 2.**
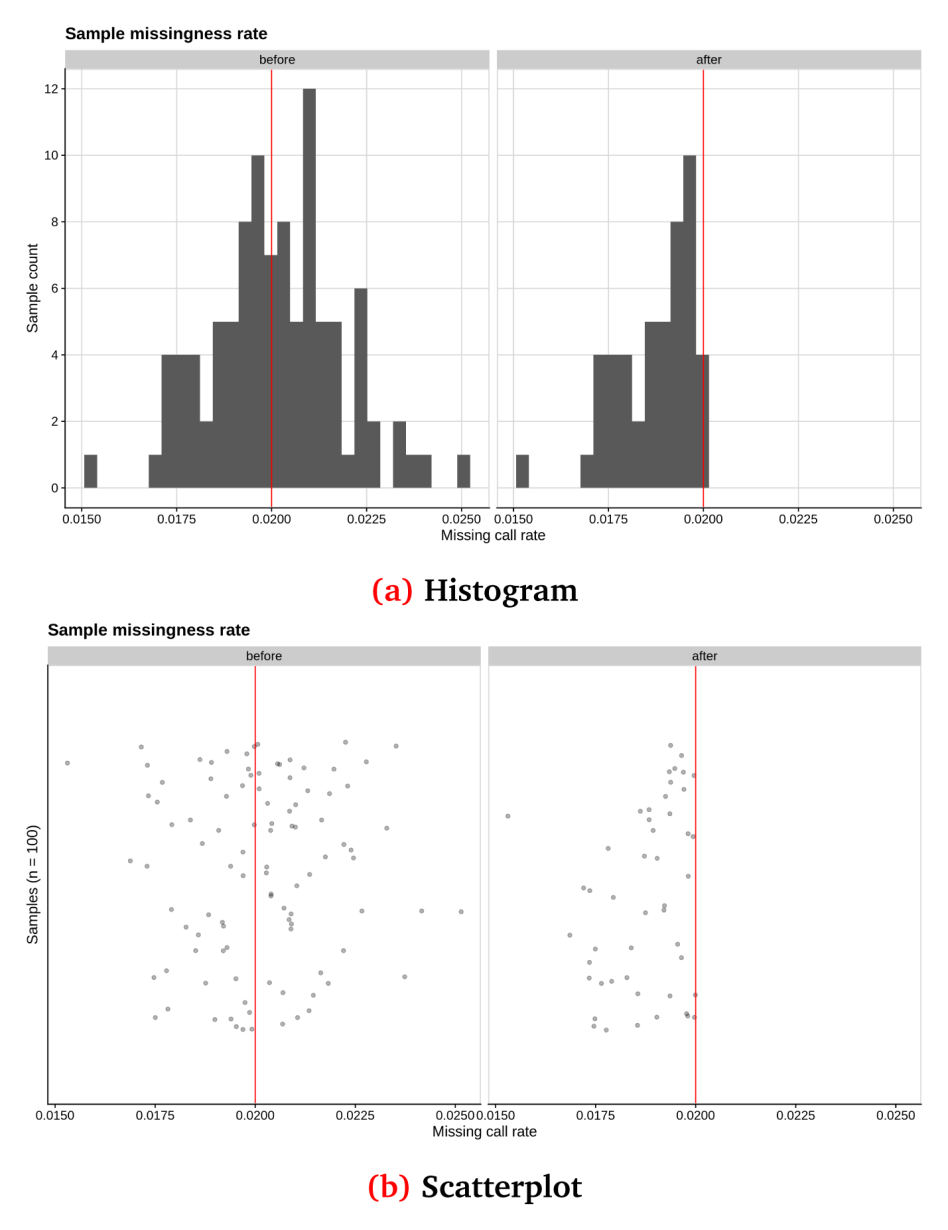
Sample call rate for synthetic toy dataset shown as ( **a**) a histogram and ( **b**) a scatterplot, before and after applying a threshold of 2% (red line). This synthetic randomised dataset was created for demonstration purposes, thus the sample call rate distribution may not closely resemble a real-world dataset.

**Figure 3.**
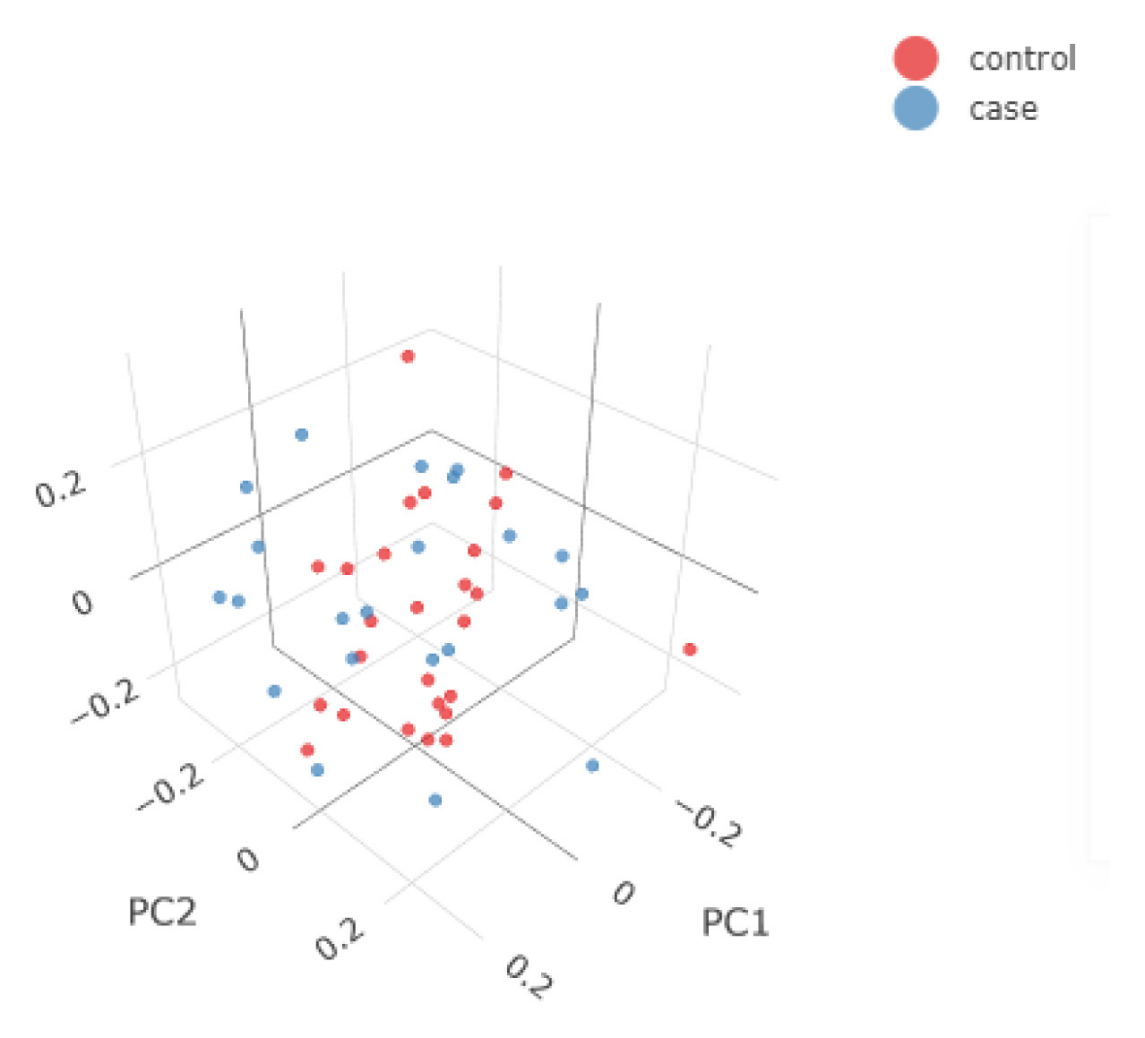
3D Principal Components Analysis (PCA) of the synthetic dataset. The samples are annotated based on their phenotype present in the input .fam file (e.g. case/control status). The 3D PCA plot is available in an interactive environment incorporated into the HTML reports. PCA plots are also provided in a 2D format for the first three Principal Components.

In Figure 2 and Figure 3, we show some examples of the output plots from the Sample and Variant QC workflows for the toy dataset. Figure 2 illustrates a sample call rate histogram and a scatterplot for the toy dataset, before and after the chosen threshold has been applied (indicated by a red line). Figure 3 shows one of the last processes of the Variant QC workflow, where Principal Component Analysis is performed on the clean dataset both for data exploration and the first X Principal Components are used as covariates in the GWAS workflow (if the user has not provided a separate covariate file), to account for a potential inner population sub-structure.

### Population stratification

The aim of the Population Stratification workflow is to identify and then remove potential outliers, based on population structure, using the EBI’s latest release of a processed phased 1,000 Genomes Project reference panel, aligned to human genome build 37. Population stratification is an essential step in QC analysis, since it minimises the possibility that the difference in the allele frequencies is caused by the different ancestry of the samples. The Population Stratification workflow requires the main QC workflows.

During this workflow, internal processing of both the 1,000 Genomes data and of the user’s dataset are performed, and then the two datasets are merged, keeping only mutual SNPs shared by both. The internal processing consists of numerous QC steps, some of which can be tailored by the user, by passing the following parameters:

--indep_pairwise: Control PLINK’s variant pruning process.
--variant_geno: Remove poorly genotyped variants.

When both datasets are prepared and merged, snpQT creates a population file labelling the ancestry of each sample. The user's samples are automatically labelled as "OWN". The population label for the 1,000 Genomes data can be controlled by the --popfile parameter, using super-population labels (e.g. EUR, AFR, AMR) or subpopulation labels using the --popfile [super, sub] parameter. When the population file and the merged dataset are ready, EIGENSOFT's smartpca software is performed for automatic outlier removal. Smartpca takes a set of parameters, which can be in the form of a file. snpQT provides the option to change this file according to the users' needs using the --parfile parfile.txt parameter. Lastly, the user can choose to infer eigenvectors based on a population subset list in smartpca using the parameter --popcode.

To perform population stratification on the toy dataset the user can run the following command:


nextflow run main.nf \
  -profile standard,singularity \
  -resume \
  -params-file parameters.yaml \
  --bed data/toy.bed \
  --bim data/toy.bim \
  --fam data/toy.fam \
  --qc \
  --pop_strat \
  --results results_toy/ \
  --sexcheck false


After successful completion, a new sub-folder pop_strat/ will be created within the –-results directory, along with the previous qc/ folder. Since the -resume parameter was used, the Sample QC processes have been cached, making the pipeline run faster. As it was mentioned above (and seen in Figure 1), Population Stratification runs in between Sample and Variant QC workflows, which means now that --pop_strat is combined with --qc, the input files for the Variant QC workflow have changed and therefore, the corresponding processes will run again with the updated input. Within the pop_strat/ folder, the following are included:

- A bfiles/ folder including three binary PLINK files and a .log file coming from the last process of the --pop_strat workflow.
- A figures/folder including six 2D plots for the first three Principal Components, two 3D plots in a .rds format for an interactive user experience for both before and after outlier removal using EIGENSOFT and lastly, two .log plots summarising the number of samples and variants in each step of the workflow.
- A logs/ folder including a pop_strat_log.txt file summarising details about the numbers of samples, variants, and phenotypes for each step of the workflow, as well as the working directory where the intermediate files for each process are stored, so that it is easier for the user to inspect the results.
- An HTML report summarising all the plots, as well as hosting an interactive environment for the 3D plots.

**Figure 4.**
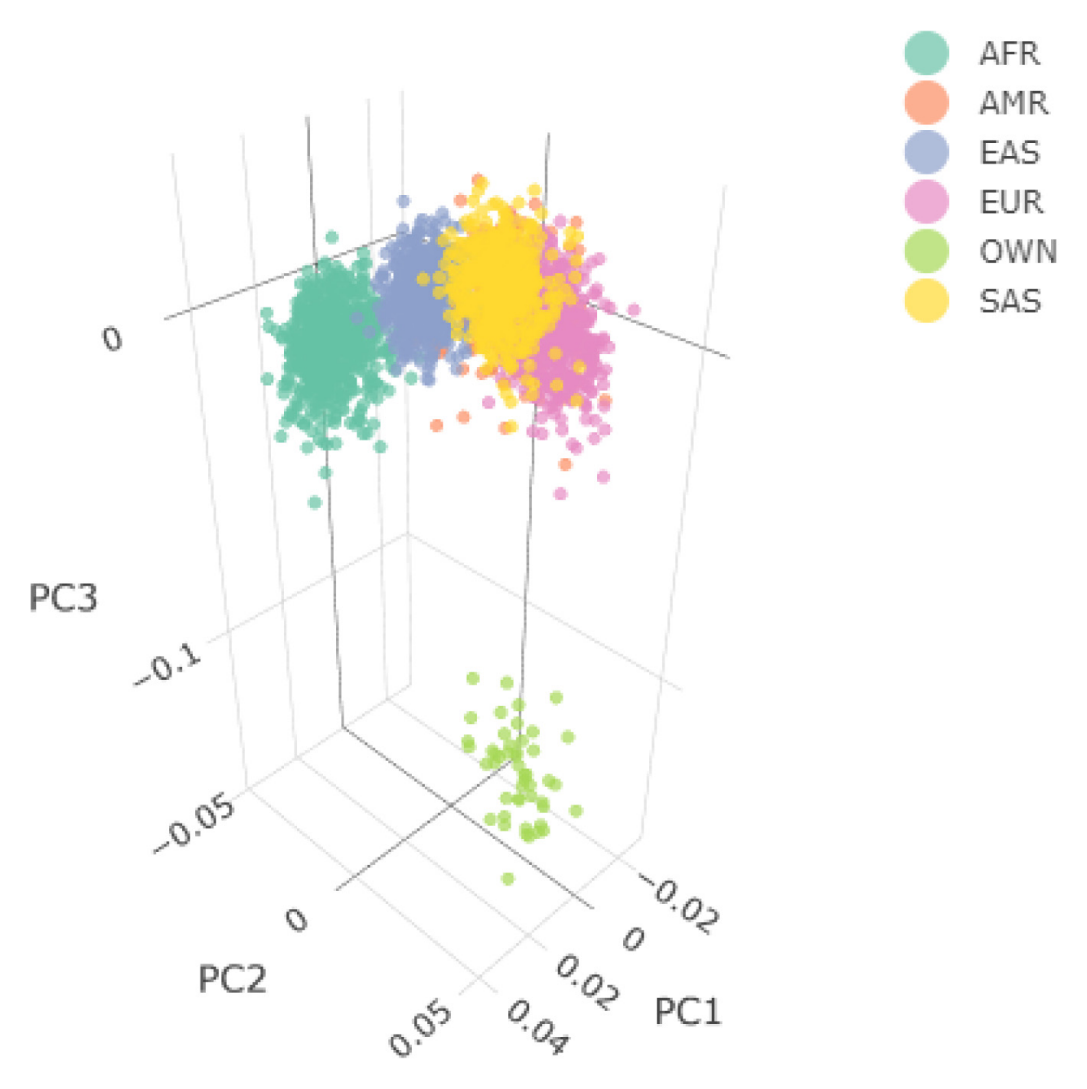
3D Principal Components Analysis (PCA) of the synthetic dataset following combination with the 1,000 Genomes Project data. The samples are annotated based on their ancestry, except for the user’s data which are labelled as "OWN". Two 3D PCA plots are available in an interactive environment incorporated in the HTML reports, representing before and after outlier removal using EIGENSOFT. PCA plots are also provided in a 2D format for the first three Principal Components. Since the synthetic toy dataset is artificial and contains only a few hundred of randomised independent markers, it is located a large distance from the 1,000 Genome data. For this reason, the before-and-after outlier removal PCA plots are identical, so only one is shown here.

Figure 4 shows the Principal Component Analysis (PCA) plot for the synthetic toy dataset. The PCA topology is quite artificial here, as this is a synthetic dataset made in PLINK2, containing only a few thousand genotypes of chromosome 1, which are subsequently pruned, leaving a few hundred independent SNPs merged with 1,000 Genomes Project data. A more natural example of PCA plots resulting from real-world data is shown in the online tutorial for the ALS dataset.

### GWAS

This workflow performs both logistic and linear regression for binary and quantitative phenotypic traits. snpQT performs a logistic regression by default (using the parameter --gwas in the terminal or setting gwas: true in the parameter file), but it is also designed to run linear regression on quantitative phenotypes using the –-linear true parameter in combination with the --gwas parameter. These analyses can be performed with and without adjusted covariates to account for a fine-scale population structure. Covariates can be calculated at the end of the --qc workflow (preferably used with the Population Stratification workflow) using the first X Principal Components of the generated PCA, using the --pca_covars parameter; alternatively, covariates can be passed directly from the user as an argument with --covar_file. The covar.txt file should follow the same format as a PLINK covariate file. The GWAS workflow requires the main QC workflow to run in advance. For a logistic regression analysis on the toy dataset, the user can run the following command (the following example assumes that the user wishes to run Population Stratification workflow as well, although this is not obligatory):


nextflow run main.nf \
  -profile standard,singularity \
  -resume \
  -params-file parameters.yaml \
  --bed data/toy.bed \
  --bim data/toy.bim \
  --fam data/toy.fam \
  --qc \
  --pop_strat \
  --gwas \
  --results results_toy/ \
  --sexcheck false


The command above causes a total of 42 separate snpQT processes to run. When GWAS has finished running successfully, a new gwas/ sub-folder will be created within the results_toy/ directory, along with the previous pop_strat/ and qc/ folders mentioned above. Within the gwas/ folder the following are included:

- A files/ folder including the PLINK2 Generalised Linear Regression results of GWAS analyses, both with and without adjusted covariates, accompanying log files and GWAS files with adjusted p-values for multiple-testing corrections.
- A figures/ folder including Quantile-Quantile (Q-Q) plots and Manhattan plots for the GWAS results, both with and without covariates, and log plots illustrating the number of samples and variants at each step.
- A logs/ folder including a gwas_log.txt file summarising details about the numbers of samples, variants, and phenotypes for each step of the corresponding workflow, as well as the working directory where the intermediate files for each process are stored, so that it is easier for the user to inspect the results.
- An HTML report summarising all the plots.

**Figure 5.**
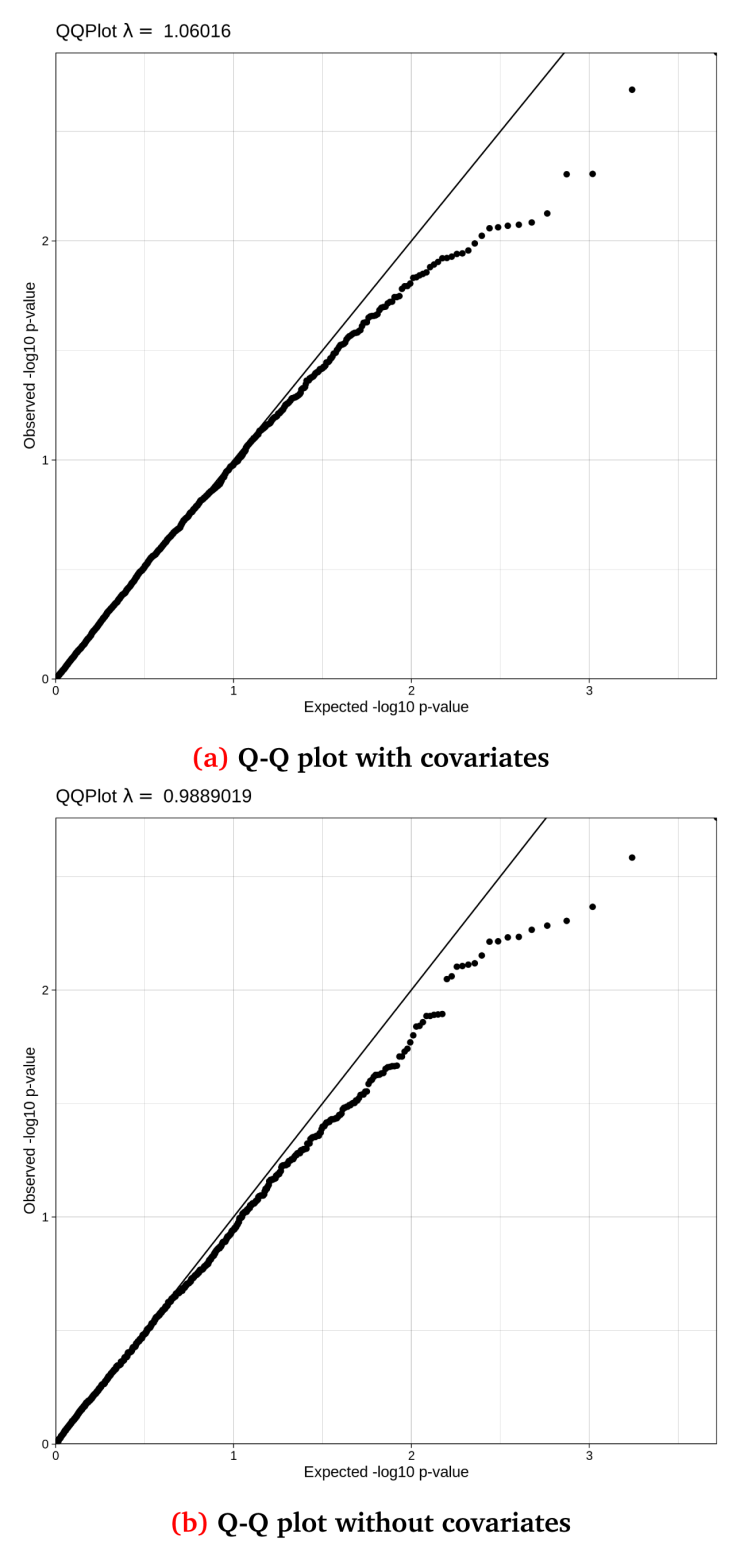
Q-Q (Quantile-Quantile) plots for the synthetic dataset both ( **a**) with and ( **b**) without covariate adjustment, with their accompanying lambda values. This synthetic randomised dataset was created for demonstration purposes, thus the Q-Q distributions may not closely resemble a real-world clean dataset.

**Figure 6.**
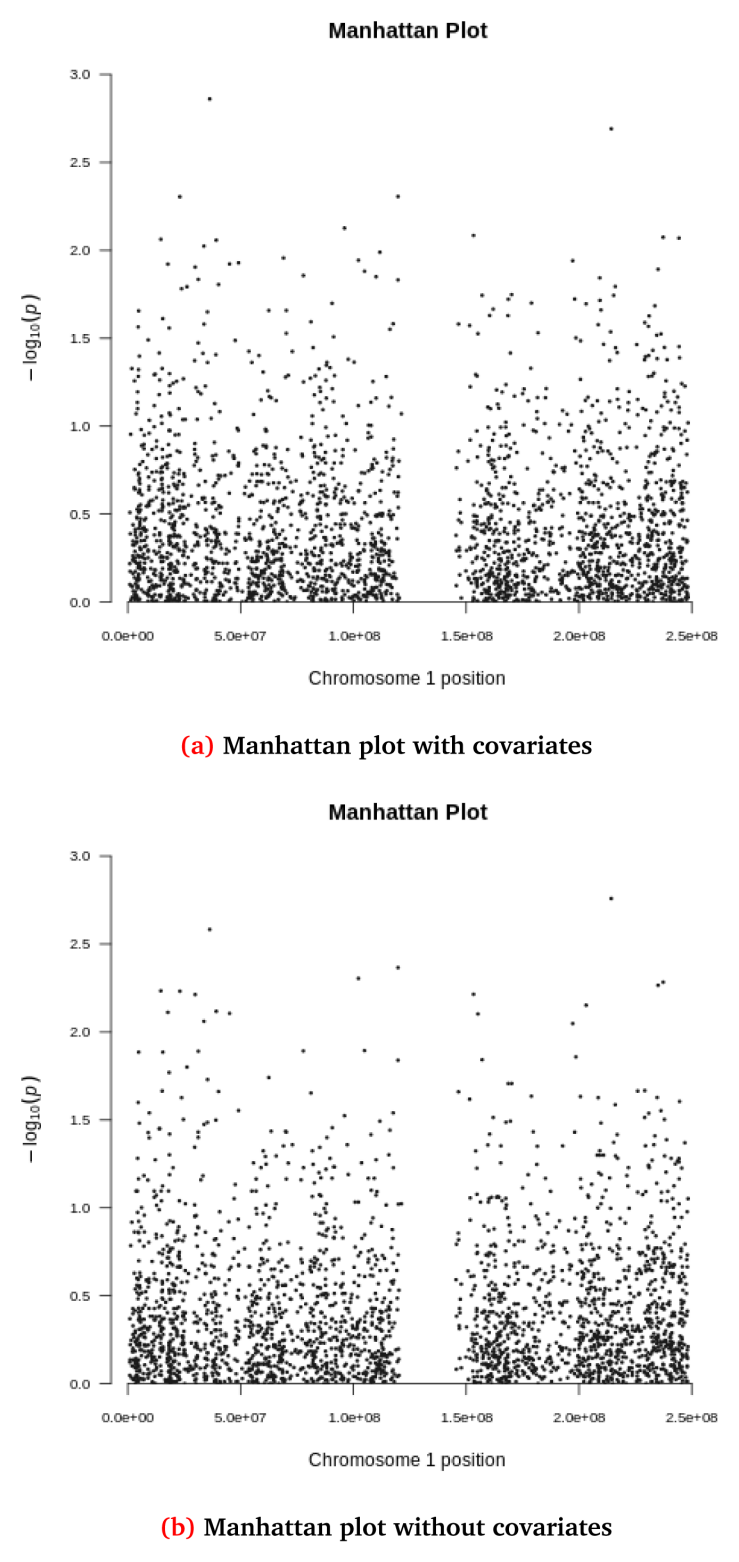
Manhattan plots for the synthetic dataset both ( **a**) with and ( **b**) without covariate adjustment. Each dot represents a variant located based on its genomic position (x-axis) and GWAS -log(p-value) (y-axis). The synthetic dataset at this stage contains 2,312 variants located in chromosome 1.

Figure 5 and Figure 6 show Q-Q and Manhattan plots, with and without covariate adjustment for the synthetic toy dataset, respectively.

On our development system, using the same workflows as in the example above (but including a sex discrepancy check and plotting, as well as a missing phenotype check, so that snpQT ran 45 processes in total), applied to the 2,000 case-control ALS cohort demonstrated in our online documentation and also to a 12,319 case-control ALS cohort (all the samples of dbGaP accession number: phs000101.v5.p1), snpQT completed in 4 minutes and in 3h 14m, respectively. Performance metrics for these two cohorts are provided ( https://doi.org/10.5281/zenodo.5703398). 

### Pre-Imputation, imputation & post-imputation

The Pre-Imputation workflow prepares a genomic dataset for phasing and imputation, including fixing issues such as flipping SNPs that are on the reverse strand, removing ambiguous and duplicated SNPs and fixing the reference allele. This workflow was designed according to the Sanger Imputation Server preparation guidelines. If the user wishes to run the Pre-Imputation workflow independently (i.e. the user is not interested in local imputation) then it is required to combine the workflow with the main QC workflow (and only optionally with the Population Stratification workflow). When Pre-Imputation QC is run independently, the --pre_impute argument is used and it cannot be combined with the GWAS, Imputation and Post-Imputation workflows. This means that it is important to set --gwas, --impute and --post_impute parameters to false (the example parameters.yaml file already has these set to false). To run Pre-Imputation QC on the synthetic dataset, the user can run the following command:


nextflow run main.nf \
  -profile standard,singularity \
  -resume \
  -params-file parameters.yaml \
  --bed data/toy.bed \
  --bim data/toy.bim \
  --fam data/toy.fam \
  --qc \
  --pre_impute \
  --results results_toy/ \
  --sexcheck false


The Pre-Imputation workflow creates a preImputation/files/ directory, which contains a compressed VCF and an indexed CSI files which are ready for phasing and imputation, compatible with the Sanger Imputation Server standards.

snpQT also offers an optional Imputation workflow, using the Pre-Imputation workflow prepares a genomic dataset parameter –-impute, where the user can increase the number of markers of their genomic dataset, using EBI’s latest release of the phased 1,000 Genomes Project reference panel aligned to human genome build 37. When the --impute workflow is used, the Pre-Imputation and Post-Imputation workflows are called internally before and after phasing, and local imputation takes place, accordingly. As explained above, Pre-Imputation prepares the user’s dataset for phasing using shapeit4 and local imputation with impute5. When phasing and imputation per chromosome are finished, then the Post-Imputation workflow takes as input the imputed chromosomes, and filters out all poorly imputed variants, based on Info score and MAF. The user can alter these filters using --info and --impute_maf parameters, respectively. The Post-Imputation workflow also annotates missing SNP IDs and handles different categories of duplicated SNPs.

To run local imputation on the synthetic toy dataset the user can run the following command:


nextflow run main.nf \
  -profile standard,singularity \
  -resume \
  -params-file parameters.yaml \
  --bed data/toy.bed \
  --bim data/toy.bim \
  --fam data/toy.fam \
  --qc \
  --pop_strat \
  --impute \
  --gwas \
  --results results_toy/ \
  --sexcheck false


When imputation is complete, separate preImputation/ and post_imputation/ folders are created. The post_imputation/ directory contains the following sub-folders:

- A bfiles/ folder including three binary PLINK imputed files and a .log file coming from the last process of the Post-Imputation workflow.
- A figures/ folder including log plots illustrating the number of samples and variants at each step.
- A logs/ folder including a post_impute_log.txt file summarising details about the numbers of samples, variants, phenotypes and the processes of the workﬂow.

Despite that the Post-Imputation workﬂow can be nested under the --impute workﬂow, it is also designed to run independently using the --post_impute parameter; as some users may prefer to run imputation on external online servers, or may have already imputed data and they wish to proceed only with a Post-Imputation QC. When the --post_impute parameter is used, it is important to set all other workflow parameters to false. When this process is not followed, snpQT will output an error message prompting the user to set incompatible workflows to false. The accepted input files for the Post-Imputation workflow are a valid imputed VCF file containing an INFO column and an accompanying PLINK .fam file, which should both contain the same samples. To run Post-Imputation QC on the synthetic toy dataset, the user can run the following command:


nextflow run main.nf \
  -profile standard,singularity \
  -resume \
  -params-file parameters.yaml \
  --vcf merged_imputed.vcf.gz \
  --fam results_toy/qc/bfiles/E11.fam \
  --post_impute \
  --qc_false \
  --results results_postImpute/


Please note that, when setting the --vcf parameter, the user will need to give the correct path to merged_imputed.vcf.gz – this will be the work/ directory of the imputation:merge_imp process that is displayed in the terminal when running the Imputation workflow. We use the .fam file from the results folder because it is important that the imputed VCF file contains the same samples as the provided .fam file.

As already explained above, the results directory contains a new post_imputation/ folder containing the same elements as in the Imputation workﬂow.

Lastly, snpQT provides a command-line help page, using the parameter --help. We provide advanced installation guides as well as further information about the implemented snpQT processes, the synthetic toy dataset and the real-world ALS dataset results in the online documentation at https://snpqt.readthedocs.io/en/latest/.

## Conclusions

The snpQT tool offers robust QC combined with scalability, reproducibility, ﬂexibility and user-friendly design which can appeal to a broad spectrum of users. It is a stand-alone software, implemented as a modular nextﬂow DSL2 workﬂow. No additional coding nor manual installation/download of any data or other program are required apart from nextﬂow and Anaconda, Singularity, Docker or Environment Modules. We have designed environments and selected specific versions of standard bioinformatics tools for each stage of the workﬂow, to ensure consistency and compatibility. The input for snpQT is a VCF file and/or binary PLINK files, formats which are widely used. The architecture of snpQT provides a thorough QC analysis (inspired and/or tested by previous authors ^11, 28, 29^), including parameters and generating plots, for both before and after the provided threshold for the majority of the steps. Outputs include interactive HTML reports, summative .log files and graphs summarising all the results for easier inspection. For users who have limited experience with QC analysis, a thorough "how-to" guide and step-by-step tutorials are provided, using the demonstration dataset that is available with the tool, which can also be informative to users who wish to be newly acquainted with QC analysis.

## Software availability

Source code available from: https://github.com/nebfield/snpQT

Archived source code at time of publication:

Zenodo: nebfield/snpQT: v0.1.7 -Fluffy penguin, https://zenodo.org/record/5682566 ^30^

License: MIT

## Data availability

### Underlying data

Zenodo: snpQT reference data (Version 0.1), http://doi.org/10.5281/zenodo.4916469 ^31^

- This project contains the processed reference data required by snpQT to function. snpQT can download and process raw data from scratch to create reference data, as described in the installation section, or the data above can be downloaded and used to save time.

Zenodo: nebfield/snpQT: v0.1.7 -Fluffy penguin, https://doi.org/10.5281/zenodo.5682566 ^30^

- This project contains the synthetic dataset used in the Use Case section, which is distributed with the source code.

Zenodo: snpQT performance metrics, https://doi.org/10.5281/zenodo.5703398

- This project contains the performance metrics for two ALS cohorts of different sizes.

NCBI dbGaP: Mega-GWAS ALS I. Accession number, phs000101.v5.p1: https://identifiers.org/dbgap:phs000101.v5.p1

- This dataset is under restricted access. To access the dataset, access must be requested through the dbGaP authorised access portal ( https://dbgap.ncbi.nlm.nih.gov/aa/wga.cgi?page=login).
