# Supplementary material for: snpQT: flexible, reproducible, and comprehensive quality control and imputation of genomic data
Source: F1000Res. 2021 Nov 29;10:567. Originally published 2021 Jul 14. [Version 2] doi: 10.12688/f1000research.53821.2 (PMC8637247; doi:10.12688/f1000research.53821.2)
Supplement: Peer review history

# Peer review: Reviewer response for version 2

**DOI:** 10.5256/f1000research.78931.r101451

**Published:** 2022-01-10

**Authors:** Andries T. Marees

**Affiliations:** 1 Department of Economics, School of Business and Economics, VU University Amsterdam, Amsterdam, The Netherlands

All my concerns have been addressed by the authors.

Are the conclusions about the tool and its performance adequately supported by the findings presented in the article?

Partly

Is the rationale for developing the new software tool clearly explained?

Yes

Is the description of the software tool technically sound?

Yes

Are sufficient details of the code, methods and analysis (if applicable) provided to allow replication of the software development and its use by others?

Yes

Is sufficient information provided to allow interpretation of the expected output datasets and any results generated using the tool?

Yes

Reviewer Expertise:

GWAS, method development, statistics, addiction, psychiatry, socio-economic status

I confirm that I have read this submission and believe that I have an appropriate level of expertise to confirm that it is of an acceptable scientific standard.

# Peer review: Reviewer response for version 2

**DOI:** 10.5256/f1000research.78931.r101450

**Published:** 2021-12-01

**Authors:** Stephanie M. Gogarten

**Affiliations:** 1 Department of Biostatistics, University of Washington, Seattle, WA, USA

The authors have fully addressed all of my concerns.

Are the conclusions about the tool and its performance adequately supported by the findings presented in the article?

Yes

Is the rationale for developing the new software tool clearly explained?

Yes

Is the description of the software tool technically sound?

Partly

Are sufficient details of the code, methods and analysis (if applicable) provided to allow replication of the software development and its use by others?

Yes

Is sufficient information provided to allow interpretation of the expected output datasets and any results generated using the tool?

Yes

Reviewer Expertise:

Human genetics, quality control, GWAS

I confirm that I have read this submission and believe that I have an appropriate level of expertise to confirm that it is of an acceptable scientific standard.

# Peer review: Reviewer response for version 1

**DOI:** 10.5256/f1000research.57243.r92711

**Published:** 2021-11-01

**Authors:** Anna Ulrich

**Affiliations:** 1 University Of Surrey, Guildford, UK

Vasilopoulou *et al.* presents a comprehensive and integrated workflow for the quality control (QC), assessment of population stratification, genome build conversion, imputation and genome-wide association (GWA) analysis of called genetic variants for those without much experience in bioinformatics. However, it seems counterintuitive to use the small synthetic dataset instead of the Amyotrophic Lateral Sclerosis (ALS) cohort (shown in the online tutorial) as a use case in the manuscript. Performance could be better demonstrated with a genome-wide dataset of a size more realistic for today`s GWA analyses. For example, the run time for the complete QC, population stratification and GWAS is presented for the synthetic dataset (~2 mins) which is not too informative for real datasets.

 In addition, I would like to point out the following statements in the text which were unclear to me:

*“When this work is run successfully, a new folder will be created named convert_build_toy/ which contains a files/ subfolder which contains three binary plink files (the .fam file contains updated phenotype information) aligned to b38 and a .vcf.gz file for users who prefer this format for other purposes.”* – The authors might have meant the updated .bim files here. It is unclear to me how the phenotype data file is updated when aligning genomic positions to a different build.

*“Remove problematic samples for which (1) pedigree sex does not agree with the predicted sex based on sex chromosome homozygosity or (2) there is no sex phenotype using plink (default mode).”* – Instead of “there is no sex phenotype using plink” the authors could specify “there is no sex phenotype present in the .fam file”, as this might be more informative to those not too familiar with the PLINK.

*“Figure 3 shows one of the last processes of the Variant QC workflow, where Principal Component Analysis is performed on the clean dataset both for data exploration and for generation of covariates using the first X Principal Components, which can then be used in the GWAS workflow, to account for a potential inner population sub-structure.”* – “generation of covariates using the first X Principal Components” would suggest that it is not the Principal Components (PC) themselves which are used as covariates in GWAS, but some other variable generated using PCs.

*“Pre-imputation workflow creates a preImputation/files/ directory, which contains a compressed VCF and an indexed VCF files which are ready for imputation." *– The authors could specify here the imputation services/servers this output is compatible with (i.e. TopMed, HRC?).

Are the conclusions about the tool and its performance adequately supported by the findings presented in the article?

Partly

Is the rationale for developing the new software tool clearly explained?

Yes

Is the description of the software tool technically sound?

Yes

Are sufficient details of the code, methods and analysis (if applicable) provided to allow replication of the software development and its use by others?

Yes

Is sufficient information provided to allow interpretation of the expected output datasets and any results generated using the tool?

Partly

Reviewer Expertise:

Statistical genetics, multi-omics

I confirm that I have read this submission and believe that I have an appropriate level of expertise to confirm that it is of an acceptable scientific standard, however I have significant reservations, as outlined above.

# Response

**Published:** 2021-11-16

**Authors:** William Duddy

**Affiliations:** University of Ulster, UK

We thank the reviewer for their time and constructive comments.

**Regarding the use of the small synthetic dataset:** The use case followed in the manuscript is intended to show how snpQT can be used. Since the ALS dataset requires an ethical process for access to it, we wished to provide a use case that users would be able to follow with minimal barriers – this is why we chose to use the synthetic dataset. However, we agree with the reviewer that it was not very relevant to refer to run time for this small synthetic dataset in the manuscript, and we have now removed reference to run time for the synthetic dataset in the new version of the manuscript. Furthermore, to give an indication of performance for datasets of more relevant size, we have now used nextflow’s built-in reporting functionality to generate performance metrics for two cohort sizes from the ALS dataset, and added this to the manuscript text:

*“On our development system, using the same workflows as in the example above (but including a sex discrepancy check and plotting, as well as a missing phenotype check, so that snpQT ran 45 processes in total), applied to the 2,000 case-control ALS cohort demonstrated in our online documentation and also to a 12,319 case-control ALS cohort (all the samples of dbGaP accession number: phs000101.v5.p1), snpQT completed in 4 minutes and in 3h 14m, respectively. Performance metrics for these two cohorts are provided ( https://doi.org/10.5281/zenodo.5703398).”*

**Regarding build conversion:** We apologise for the ambiguity and thank the reviewer for spotting this. The phenotype information is not changed by the build conversion – the word ‘updated’ referred to the technical need to preserve phenotype information by updating the PLINK files that are generated during the build conversion process, in order to ensure consistency with downstream workflows. The required input files for the human genome build conversion workflow are a VCF and a .fam file. After the LiftOver process is complete, snpQT then creates 3 binary PLINK files, using the new VCF file that has been created during the LiftOver process. Although a .fam file is generated by PLINK at this stage, it is based on the VCF file and, as VCF files do not store any phenotype information, the phenotype column in the .fam file of the previously generated binary PLINK files would be -9 i.e. missing. For this reason, the user-provided .fam file is needed to update the PLINK files with the original phenotype information. In order to avoid confusion for readers, we have changed the text simply to, *“When this work is run successfully, a new folder will be created named convert_build_toy/ which contains a files/ sub-folder with three binary PLINK files (with updated genomic information, and phenotypic information preserved).”*

**Regarding sex phenotypes: **We thank the reviewer for spotting this, and have changed the text as requested: *"Remove problematic samples for which (1) pedigree sex does not agree with the predicted sex based on sex chromosome homozygosity or (2) there is no sex phenotype present in the .fam file"*

**Regarding principal components:** Thank-you for spotting this. The text now reads, *"Figure 3 shows one of the last processes of the Variant QC workflow, where Principal Component Analysis is performed on the clean dataset both for data exploration and the first X principal components are used as covariates in the GWAS workflow (if the user has not provided a separate covariate file), to account for a potential inner population sub-structure."*

**Regarding imputation:** The text now reads *"The Pre-Imputation workflow creates a preImputation/files/ directory, which contains a compressed VCF and indexed VCF files which are ready for phasing and imputation, compatible with the Sanger Imputation Server standards.”*, and we have added similar explanation at the start of the Pre-Imputation, imputation & post-imputation section.

# Peer review: Reviewer response for version 1

**DOI:** 10.5256/f1000research.57243.r91453

**Published:** 2021-08-26

**Authors:** Stephanie M. Gogarten

**Affiliations:** 1 Department of Biostatistics, University of Washington, Seattle, WA, USA

The snpQT software is a useful resource for researchers working on Genome-Wide Association Studies (GWAS). It bundles a wide collection of software together and provides a clearly defined workflow based on best practices in GWAS quality control. It will be particularly useful for beginning researchers as it provides a straightforward entry point to learning how to work with GWAS data. The software is open source and comes with extensive documentation and tutorials, all available on the web.

 The manuscript is well written and clearly describes the use of the software and its benefits, with a few caveats as outlined below:

**Major:**

 1) The term "racefile" as an argument name is inappropriate and should be changed. Race refers to a social category that is correlated with, but not the same as, genetic ancestry, and using it in this way goes against current recommendations in the human genetics community (Brothers, Bennett and Cho 2021 ^1^; Birney *et al *2021 ^2^). In fact, the allowed values in the "racefile" appear to be 1000 Genomes population labels, which are not races. A more appropriate term for this argument would be "popfile", with the associated argument "racecode" instead called "popcode".

 2) Similarly, the term "ethnic outliers" should also be changed, both in the manuscript and in the snpQT online tutorial. Ethnicity is also a social category and its use is not appropriate in this context. The outlier removal performed by EIGENSOFT does not rely on any labelling of race, ethnicity, or population, but is instead described in the original publication (Price *et al* 2006 ^3^) as "Outliers were defined as individuals whose ancestry was at least 6 standard deviations from the mean on one of the top ten inferred axes of variation." Outliers are defined with respect to mathematically inferred clusters, so using the term "ethnic" to describe outlier removal misrepresents the behavior of the underlying software. Replacing the term "ethnic outliers" with simply "outliers" would correct this problem, as the term is always presented in the context of the population structure workflow. "PCA outliers" would be another alternative.

**Minor:**

 3) "Dependency hell" is a colloquial phrase that may be confusing to many readers. Please use a longer sentence in the abstract to more clearly describe the issue. The methods section already has such a description and the same text can be used in the abstract. Remove the words "known as 'dependency hell'" from the methods section, as it is unnecessary and the choice of words could potentially alienate some readers.

 4) The description of the installation process in the manuscript seems to be out of date when compared with the online tutorial. Although it is expected that software is updated after manuscript publication, during the publication and review process the manuscript should reflect the current operation of the software. Specifically:

- The manuscript provides commands to download the development version of the software, while the tutorial recommends an alternative method to get the latest stable release.
- Links for tar.gz files with reference data are out of date.
- The manuscript references only Anaconda and Docker as options for running the software, while the tutorial recommends Singularity over Docker. 

5) The authors should consider changing the default behavior of the build conversion to be build 37 to 38. As a user, the behavior I would expect (and desire) would be converting to the most current build by default. The authors do provide rationale for their choice, citing that public reference panels are available in build 37. However, 1000 genomes data is currently available in build 38, so the reference dataset could be updated.

Are the conclusions about the tool and its performance adequately supported by the findings presented in the article?

Yes

Is the rationale for developing the new software tool clearly explained?

Yes

Is the description of the software tool technically sound?

Partly

Are sufficient details of the code, methods and analysis (if applicable) provided to allow replication of the software development and its use by others?

Yes

Is sufficient information provided to allow interpretation of the expected output datasets and any results generated using the tool?

Yes

Reviewer Expertise:

Human genetics, quality control, GWAS

I confirm that I have read this submission and believe that I have an appropriate level of expertise to confirm that it is of an acceptable scientific standard, however I have significant reservations, as outlined above.

## References

[1] : Taking an antiracist posture in scientific publications in human genetics and genomics. *Genet Med* .23(6) : 10.1038/s41436-021-01109-w 1004-1007 10.1038/s41436-021-01109-w 33649579

[2] : The language of race, ethnicity, and ancestry in human genetic research. *arXivLabs* .2021;

[3] : Principal components analysis corrects for stratification in genome-wide association studies. *Nat Genet* .2006;38(8) : 10.1038/ng1847 904-9 10.1038/ng1847 16862161

# Response

**Published:** 2021-10-29

**Authors:** William Duddy

**Affiliations:** University of Ulster, UK

1. We thank the reviewer for noting this very important oversight, and apologise for the misuse of the terminology. We have updated our code, documentation and manuscript, changing the parameters' names from –racefile to –popfile, --racecode to –popcode.
2. Again, we thank the reviewer for noting this very important oversight. Accordingly, we have removed the adjective "ethnic" from our online documentation and manuscript. As the reviewer suggested, we now refer to the excluded samples after population stratification as "outliers".
3. We have changed the abstract from "Dependency hell and reproducibility are recurrent challenges" to "Software incompatibilities, and inconsistencies across computing environments, are recurrent challenges, leading to poor reproducibility". We also removed the term "dependency hell" from the methods.
4. We apologise for this - we have been improving the installation procedure, as described in response to reviewer 1, and during this process inconsistencies arose between the manuscript and the documentation. We have now updated both the manuscript and the online documentation with our latest instructions.
5. We made a decision early in the development of snpQT to focus on build 37, as this is still the most commonly used reference. As such, build 37 is the primary build for the current release that is described in the manuscript, and considerable effort has been expended to ensure that the tool functions well with this build. We are working to support build 38, but this is likely to be included in a future version of snpQT, and as such is beyond the scope of the current work.

# Peer review: Reviewer response for version 1

**DOI:** 10.5256/f1000research.57243.r89658

**Published:** 2021-08-11

**Authors:** Andries T. Marees

**Affiliations:** 1 Department of Economics, School of Business and Economics, VU University Amsterdam, Amsterdam, The Netherlands

This paper by Vasilopoulou *et al.* is well written and presents a potentially interesting software pipeline for genomic qc. Furthermore, the accompanying website provides a clear overview of all steps and options. 

**Major concerns:**

 1A) The authors write: “The analyst may encounter incompatibility problems and installation difficulties as well as spending valuable time familiarizing themselves with a number of different tools that sometimes lack detailed documentation”.

 I agree with the authors that this is an issue the field is faced with and see the value snpQT can offer. Unfortunately, the snpQT installation difficulties (Anaconda/Docker/modules) are on par with many other packages. I strongly recommend looking into how you can make this process easier (if possible, aim for full copy-paste simplicity). Especially because the target audience is probably inexperienced analysts/researchers. I believe, based on my experience in providing tutorials/software tools that, unfortunately, 90% of potential users won’t make it past the current installation process.

 1B) A strong selling point of this paper is that all steps from QC, to imputation, to the results including figures, are all under one umbrella, which is great.

 However, many potential users will most likely use an HPC and have no root access for a docker installation. The authors write that this is needed for the imputation part as this is not possible with Anaconda. If the imputation might not be available for many users the question arises, what is the added benefit of this software pipeline? Clear protocols for all the separate steps (QC, imputation, association) are already widely available (and already in place for established groups).

 I am aware that the above problem can be solved using environment modules (as the authors write), but the current paper/website leaves the potential users on their own to figure the rest out. I am afraid that these hurdles will strongly limit adoption of this otherwise promising software package. 

 2) Personally I do not like the defaults with the --qc command for example, and believe it is better for the user to specify input for the various qc steps. The default thresholds provided by the authors are not in all data sets per se the ideal choices (e.g., 3 PC as covariates or MAF 0.05). I see no benefit for the defaults but do see them causing potential negative outcomes - does snpQT provide the user with the flexibility to choose the order of the QC steps or is the sequence of steps hardcoded? (i.e., flexibility in order of the steps within and between the various workflow parts).

Are the conclusions about the tool and its performance adequately supported by the findings presented in the article?

Partly

Is the rationale for developing the new software tool clearly explained?

Yes

Is the description of the software tool technically sound?

Yes

Are sufficient details of the code, methods and analysis (if applicable) provided to allow replication of the software development and its use by others?

Yes

Is sufficient information provided to allow interpretation of the expected output datasets and any results generated using the tool?

Yes

Reviewer Expertise:

GWAS, method development, statistics, addiction, psychiatry, socio-economic status

I confirm that I have read this submission and believe that I have an appropriate level of expertise to confirm that it is of an acceptable scientific standard, however I have significant reservations, as outlined above.

# Response

**Published:** 2021-10-29

**Authors:** William Duddy

**Affiliations:** University of Ulster, UK

We thank the reviewer for their time and very constructive comments.

 1A) We apologise if the reviewer had problems installing snpQT, and we have taken further steps, detailed below, to make this as straightforward as possible. We would like to note in addition that, whereas a user may be faced with installation challenges and version choices for multiple separate tools if building their own ad-hoc analytical pipeline, snpQT requires only a single installation and compatible software versions are pre-selected. 

 We have now simplified our Quickstart online documentation guide, aiming, as the reviewer suggested, for "full copy-paste simplicity" insofar as possible. We have updated our initial installation guides, adding further simple installation details about the provided profiles (Anaconda/Singularity/Docker/Modules) in a dedicated "Profiles" tab, and moving the more advanced installation details to a later subsection of the User Guide. Installation now contains only the necessary basic information for a new inexperienced user to get started with snpQT, including a subsection containing set up guidelines for users who wish to perform local imputation, and quick examples on how to run snpQT. Once their profile is created the user should not need to worry about any software inconsistencies, as the selected environments will combine specific software versions selected and tested for compatibility. In addition, environment setup has been streamlined: instead of having to wait for a large environment recipe to be solved before any process runs, the appropriate environments for each workflow combination are now created while each snpQT process is running.

 1B) We thank the reviewer for the very positive comment. We agree that the need for root access had been a frustrating limitation. We have worked to overcome this, and now provide four different profile options including Anaconda, Singularity, Docker, and Environment Modules, to enable users to run the software in multiple environments, and now avoiding a requirement for root access to run on HPC. We provide two different options that enable the users to run snpQT on HPC, which are Singularity and Environment Modules. We apologise that our online documentation lacked some details on the profile installation, and we have now updated our installation guides (as mentioned above) and we further provide details and example code on how users who are interested in running their experiments on HPC can now submit to a SLURM queue.

 In general, we have worked to simplify the way snpQT sets an environment. We now provide two different types of profiles; the first type concerns the machine where snpQT is expected to run, i.e. a user working in a normal laptop would use -profile standard and in an HPC -profile cluster. The second type is responsible to set up successfully all the required libraries and software, assuming that the user has not installed anything except nextflow and the profile environment software of choice (e.g. Singularity, Anaconda, Docker). These two different types of profiles are expected to be combined according to the user’s needs and expectations. For instance, a user who would be interested to run their experiments in a HPC would use -profile cluster, singularity or -profile cluster,modules. Submission to a SLURM queue can be achieved by editing the cluster.config file in our “Profiles” tab in the online documentation at https://snpqt.readthedocs.io/en/latest/quickstart/profiles/

 2) We thank the reviewer for questioning the use of defaults – we had felt on balance that it would enhance user-friendliness to enable defaults while simultaneously providing the flexibility to change them, but we are fully aware that each analysis is unique so user needs and preferences can (and should) differ widely. To avoid these concerns, we have now removed defaults so that snpQT will not run when a necessary parameter is not specified. In addition, the reviewer’s comment prompted us to reflect that parameters specified through the command line can be at risk of mistaken or accidental misuse use through on-the-fly copy/pasting. To combat this risk, we have now implemented a parameter file that can be used in place of command line flags, and we now recommend this as the primary way to use snpQT.

 With respect to the wider query on flexibility to choose the order of QC steps, this consideration was a central concern in the design of snpQT. The pipeline is designed to offer multiple combinations of workflows (as shown in Figure 1), and the quality control steps are implemented with numerous user-modifiable thresholds. In fact, we have preserved, to the extent logical, all of the modifiable parameters of each of the external tools incorporated by the workflows, so that some 36 parameters are modifiable by the user (within the accepted range of values which is provided by the external tools). In Figure 1, we show how all 9 implemented workflows can be combined in different ways. As far as the processes are concerned, there is a specific order which they are expected to follow, based on previously established QC protocols; however, we have implemented special parameters which can be used to skip certain processes and adjust the pipeline to the user’s needs. We have adjusted the manuscript text in several places to further highlight the flexibility of snpQT.
